# Service and Silence

**Published:** 1915-05-15

**Authors:** 


### SERVICE AND SILENCE.

In these columns we naturally make no pretence to criticise or to examine the policy or conduct of those charged with international actions and responsibilities. Such questions have to be tested before other tribunals and are to be judged after a survey of all the circumstances. It happens, however, that at the moment there is a much-debated point which seems, on professional grounds, to have a special appeal to members of the medical profession. We refer to the discussion relative to the publication of details of the fighting and to the exclusion of newspaper correspondents from the area of conflict. There are many considerations bearing on these proceedings which do not concern us here, and upon the value of these we say nothing, either in one direction or the other. What we have to say on the matter relates purely to an aspect of the issue in which professional sympathies may be said to be particularly engaged.

The criticism which is most frequently applied to the policy of silence claims that this policy is objected to by the general public. It is said to lessen the interest of the nation and to chill its spirit. There is, however, another point of view to be considered—namely, that of the soldiers, and particularly of those who have to accept large responsibilities and to take decided action often involving serious risks. Here is a consideration which may be specially appreciated by members of the medical profession. They, too, though in much less dangerous circumstances and concerned with much narrower interests, have to take decisions which rest not infrequently on probabilities rather than on certainties; and the reasons which guide their actions are often technical and incapable of appreciation by the popular judgment. It would surely create something like dismay in the professional ranks were these decisions, and the reasons which prompt them, to be canvassed in the public prints by able editors and descriptive reportors. Censures by an incompetent court are not easy to bear; and still more, the risk of them is not likely to promote either clear thinking or courageous action. The phlegmatic might remain unmoved, but most of us would prefer to avoid the trial.

If condemnation by an uninformed judgment is bad, praise from such a source is still worse. With what countenance and composure could the medical profession face the prospect of the application of the leading article, the flaming headline, and the laudatory paragraph to the balanced judgment or technical action of the individual physician or surgeon? To shrink from such a discipline is the natural feeling of any man who is conscious of the necessary limitations of his own knowledge and is aware that even in his most careful calculations there is often a factor which he cannot fully command. When explanation is of no avail the fulsome compliment based on ignorance may sometimes have to be suffered; but who would wish it flaunted in the face of the public? What is true of the profession of medicine in this respect is doubtless true of the profession of arms. Larger interests may command the decision, and in such circumstances private feelings may have to yield, but whether newspaper publicity brings praise or blame our own professional experiences prompt a sincere sympathy for the individual soldiers who become its victims.

Some of our lay contemporaries since the war began have exhibited failings which call for a change in editorial methods. John Delane taught that it was the essential duty of an editor to control his paper in all departments. Several modern editors do not follow Delane's teaching, and the consequences to these newspapers are greatly lessened authority and an increasing diminution of public confidence.

